# Maintenance of Paternal Methylation and Repression of the Imprinted *H19* Gene Requires MBD3

**DOI:** 10.1371/journal.pgen.0030137

**Published:** 2007-08-17

**Authors:** Kimberly J Reese, Shu Lin, Raluca I Verona, Richard M Schultz, Marisa S Bartolomei

**Affiliations:** 1 Department of Cell and Developmental Biology, University of Pennsylvania School of Medicine, Philadelphia, Pennsylvania, United States of America; 2 Department of Biology, University of Pennsylvania, Philadelphia, Pennsylvania, United States of America; University of Cambridge, United Kingdom

## Abstract

Paternal repression of the imprinted *H19* gene is mediated by a differentially methylated domain (DMD) that is essential to imprinting of both *H19* and the linked and oppositely imprinted *Igf2* gene. The mechanisms by which paternal-specific methylation of the DMD survive the period of genome-wide demethylation in the early embryo and are subsequently used to govern imprinted expression are not known. Methyl-CpG binding (MBD) proteins are likely candidates to explain how these DMDs are recognized to silence the locus, because they preferentially bind methylated DNA and recruit repression complexes with histone deacetylase activity. MBD RNA and protein are found in preimplantation embryos, and chromatin immunoprecipitation shows that MBD3 is bound to the *H19* DMD. To test a role for MBDs in imprinting, two independent RNAi-based strategies were used to deplete MBD3 in early mouse embryos, with the same results. In RNAi-treated blastocysts, paternal *H19* expression was activated, supporting the hypothesis that MBD3, which is also a member of the Mi-2/NuRD complex, is required to repress the paternal *H19* allele. RNAi-treated blastocysts also have reduced levels of the Mi-2/NuRD complex protein MTA-2, which suggests a role for the Mi-2/NuRD repressive complex in paternal-specific silencing at the *H19* locus. Furthermore, DNA methylation was reduced at the *H19* DMD when MBD3 protein was depleted. In contrast, expression and DNA methylation were not disrupted in preimplantation embryos for other imprinted genes. These results demonstrate new roles for MBD3 in maintaining imprinting control region DNA methylation and silencing the paternal *H19* allele. Finally, MBD3-depleted preimplantation embryos have reduced cell numbers, suggesting a role for MBD3 in cell division.

## Author Summary

Genomic imprinting is a specialized system of gene regulation whereby only one copy of a gene is used, either the maternal or the paternal copy. Misregulation of imprinting in humans results in developmental disorders such as Beckwith-Wiedemann Syndrome, and is implicated in many cancers. Study of imprinted genes in mice can lead to a greater understanding of these diseases as well as insight into gene regulation. Many imprinted genes are associated with methylation on the silenced allele. The imprinted gene *H19* is maternally expressed and paternally methylated in a region upstream of the promoter known as the differentially methylated domain. This region is required for proper imprinted expression of *H19* and its upstream imprinted neighbor *Igf2*. Our studies have explored the requirement for methyl-CpG binding protein 3 (MBD3) in silencing of the paternal allele. MBD3 is known to be part of a repressive complex that resides at silenced genes. In our experiments, we have shown that MBD3 is required for imprinting of *H19,* and is also required for the maintenance of methylation on the paternal allele. Finally, the MBD3 protein can be found at the differentially methylated domain. The identification of a protein required for silencing of the paternal allele of *H19* is an important step in understanding regulation of this gene.

## Introduction

Genomic imprinting, an epigenetic process resulting in expression of one parental allele, is an important mechanism of transcriptional control in mammals [1,2]. Failure of transcriptional regulation defines the molecular basis for many human diseases, emphasizing the importance of this control for normal growth and development. Accordingly, loss of imprinting is implicated in a number of human diseases and cancers. For example, Beckwith-Wiedemann syndrome, a disorder characterized by overgrowth and tumor development, results from defects in gene expression from either of two linked but independently controlled imprinted gene clusters on 11p15.5, the *H19*/*IGF2* or *KCNQ1OT1*/*CDKN1C* clusters [3].

The mechanism by which imprinted gene expression is established and maintained has been extensively studied but still remains incompletely understood. DNA methylation represents one of the best candidates for conferring parental-specific expression patterns because DNA methylation is differentially acquired in the parental germlines, maintained following fertilization, and subsequently employed to silence the nonexpressed allele. One of the best examples of such regulation is observed at the mouse *H19*/*Igf2* locus [4]. The *H19* gene, which encodes a noncoding RNA, and the *Igf2* gene, which encodes a fetal mitogen, are expressed from opposite parental alleles, but their imprinted expression is regulated by common DNA elements [5–7]. One such element is a 2-kb imprinting control region located 2 kb upstream from the *H19* transcriptional start site, designated the differentially methylated domain (DMD), which is required for imprinted expression of both *H19* and *Igf2* [8]. The DMD forms an active CTCF-dependent insulator on the maternal allele that governs expression of *H19* and repression of *Igf2*. In the male germline, the DMD acquires methylation that is necessary for repression of the paternal *H19* allele [9–13]. Methylation of the DMD is essential for imprinted expression because *H19,* which is normally maternally expressed throughout development, is biallelically expressed in DNA methyltransferase 1 *(Dnmt1)* mutants [14]. Thus, while much is known about the sequences required for *H19* imprinting and the necessity for differential DNA methylation at this locus, less is known about how DNA methylation leads to silencing of the paternal allele.

Although CTCF has been shown to bind the unmethylated DMD on the maternal *H19* allele, leading to formation of an insulator, cofactors that are required for recognition of the repressed paternal allele have not been identified. The methyl-CpG-binding proteins (MBDs) are possible candidates for the dual jobs of recognizing DNA methylation marks and silencing transcription from the locus [15]. Biochemical studies have demonstrated that these proteins can bind methylated DNA and associate with repressive complexes in vitro. These proteins may, therefore, provide the link between DNA methylation and transcriptional silencing at imprinted loci.

To determine the role of MBDs in imprinted gene regulation, we have taken an RNAi approach to generate hypomorphic alleles. Although we and others have found that transcripts for all *Mbd* genes are present in early mouse embryos ([16]; KJR, unpublished data), *Mbd3* was chosen as the first target for RNAi because *Mbd3* null embryos die early in embryogenesis, suggesting a critical role in development [17]. Additionally, we have determined that MBD3 is bound to the *H19* DMD. Using both injections of one-cell embryos with double-stranded RNA (dsRNA) against Mbd3 (dsMbd3) and a transgenic (TG) RNAi approach that reduces Mbd3 mRNA in oocytes, we demonstrate that *H19* is activated on the normally methylated and repressed paternal allele in blastocysts with reduced amounts of MBD3 protein. This biallelic *H19* expression in Mbd3 RNAi embryos is significantly different from control embryos, which retain monoallelic expression. Other imprinted genes analyzed in the Mbd3-depleted embryos are monoallelically expressed, suggesting each locus may require a separate MBD partner. Interestingly, Mbd3 RNAi embryos have reduced DNA methylation at the *H19* locus, but not at the imprinted *Snrpn* locus, indicating a critical role for MBD3 in maintaining paternal methylation marks at the *H19* locus during a critical window of genome wide demethylation. Finally, reducing MBD3 levels affects cell division and, consequently, the size of RNAi embryos. These findings support the hypothesis that MBD proteins are required for the interpretation and maintenance of allele specific methylation marks leading to repression of the paternal *H19* allele.

## Results

### Temporal and Spatial Pattern of Expression of Mbd3 RNA and Protein during Preimplantation Development

**Figure 1:**
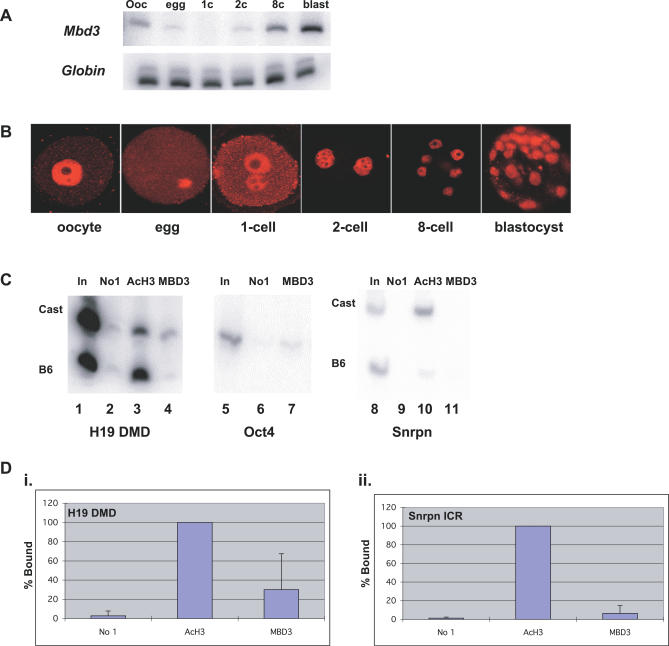
Mbd3 RNA and Protein Patterns in Oocytes and Preimplantation Embryos (A) Mbd3 and globin primers were used to amplify Mbd3 and exogenously added globin RNA from oocyte and preimplantation embryo cDNA samples. Mbd3 RNA was normalized against exogenously added globin RNA. (B) Immunocytochemistry using an anti-MBD3 antibody and a Cy3 secondary antibody showed that MBD3, a nuclear protein, is also maternally contributed to oocytes and follows a pattern similar to that of Mbd3 RNA, increasing to high levels at the blastocyst stage. (C) Chromatin immunoprecipitation (ChIP) from hybrid ES cells at the *H19* DMD, *Oct4,* and *Snrpn* loci with acetylated H3 antibody (AcH3) (Lanes 3 and 10) and MBD3 antibody (Lanes 4, 7, and 11). The *H19* DMD and *Oct4* are precipitated with the MBD3 antibody. Input chromatin (Lanes 1, 5, and 8) and no primary antibody (Lanes 2, 6, and 9) are included as positive and negative PCR controls. In the experiment presented here, 65% of the chromatin associated with the MBD3 antibody was from the paternal allele (C). (D) Quantitative real-time PCR was conducted using primers specific to the *H19* DMD (i) or to the *Snrpn* imprinting control region (ii) for antibody-precipitated samples and no primary antibody (No 1) controls. The average percentage (+/− standard deviation) of bound material for three or more chromatin preparations is shown. The AcH3 results were set at 100% and the other values were normalized to the AcH3 results. A significant enrichment of MBD3 at the *H19* DMD was evident when compared with the no-primary control (*p* < 0.05), but this enrichment was not observed at the *Snrpn* imprinting control region (*p* = 0.12). There was a significant enrichment for AcH3 histones at both loci (*p* < 0.01).

RT-PCR experiments using *Mbd3* specific primers demonstrated that Mbd3 RNA is present in oocytes and preimplantation mouse embryos (Figure 1A). The RNA was quantified and normalized relative to known amounts of rabbit globin RNA added prior to RNA preparation. Mbd3 RNA levels are high in the oocyte, become reduced through early stages of preimplantation embryogenesis, but rise again by the blastocyst stage. This profile is consistent with expression of many other transcripts; degradation of maternal transcripts followed by replacement at the two-cell stage with zygotic transcripts. MBD3 protein follows a similar distribution, with levels highest in the oocyte and blastocyst (Figure 1B). Interestingly, MBD3 protein is present on the DNA and spindle in eggs, and is nuclear at all other stages with punctate foci of stronger staining. As proposed for transcription factors that remain associated with chromosomes during mitosis and direct transcription following entry into interphase, MBD3′s association with chromatin in eggs may provide a similar molecular memory to ensure appropriate marking of the maternal allele. Thus, both Mbd3 RNA and protein are present at this critical period for imprinted gene expression when imprints are becoming established.

### MBD3 Is Bound Preferentially to the Paternal Allele at the *H19* DMD

To determine if MBD3 is associated with the *H19* locus, chromatin immunoprecipitation (ChIP) experiments were performed with an MBD3 antibody on C57BL/6 X B6(CAST7P12X) hybrid embryonic stem (ES) cells (Joanne Thorvaldsen, MSB, unpublished data). After immunoprecipitation of chromatin, PCR analysis showed that MBD3 was associated with the *H19* DMD, with a slight preferential association with the methylated paternal allele (Figure 1C). An antibody against acetylated histone H3 (AcH3) was used as a control because the 3′ end of the DMD was reported to associate with acetylated histone H3 on the maternal allele [18]. The region of the DMD reported here showed a similar preference for the maternal allele (RIV and MSB, unpublished data). Furthermore, MBD3 associates with the *Oct4* locus in undifferentiated ES cells [19], a finding we also observed, therefore validating our immunoprecipitation results. Interestingly, MBD3 was not associated with either allele of the imprinted *Snrpn* gene (Figure 1C), suggesting a specific role for MBD3 at the *H19* locus. To confirm that these results were different from no-primary controls, quantitative real-time PCR was performed at both the *H19* DMD and the *Snrpn* imprinting control region for three or more independent ChIP experiments (Figure 1D). At the *H19* locus, MBD3 was significantly associated with the locus when compared to the no-primary ChIP samples (*p* < 0.05). At the *Snrpn* locus, however, we did not see a significant association when compared to the control samples (*p* = 0.12). Although MBD3 was reported to be unable to bind directly to methylated DNA because of amino acid substitutions in its MBD domain, our results, along with MBD3 ChIP results at other loci [19,20] suggest that MBD3 is associated with DNA at repressed loci. Our data showing that MBD3 is expressed early during development, along with these ChIP experiments that demonstrate that MBD3 is bound at *H19,* provide compelling evidence for a role for MBD3 at the *H19* locus in preimplantation embryos.

### Use of RNAi for Specific Knockdown of Mbd3 RNA and Reduction in MBD3 Protein

**Figure 2:**
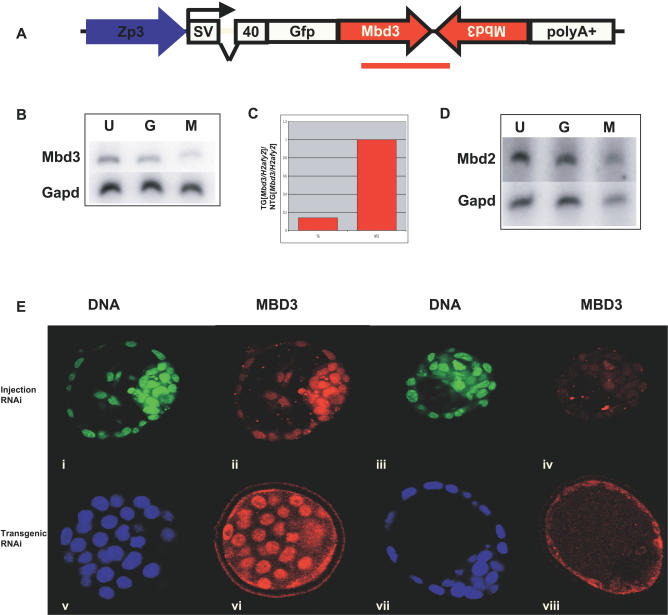
Mbd3 RNA and Protein Are Specifically Reduced in RNAi-Treated Embryos (A) A schematic depicting the RNAi transgene showing the *Zp3* promoter driving expression of *Gfp* and the *Mbd3* inverted repeat in growing oocytes. The dsRNA used in the injection RNAi experiments is designated by the red line below the inverted *Mbd3* repeat and is identical to the sequence used in the TG RNAi. (B) RNA was collected from embryos injected with dsMbd3 [M], dsGfp [G], or uninjected [U], cultured for 96 h, and collected in pools of six blastocyst-stage embryos. The RNA was used for quantitative RT-PCR. When normalized to Gapd RNA levels and compared to uninjected and dsGfp-injected control embryos, dsMbd3-injected embryos show reduced levels of RNA. The embryos shown have only 24% of the Mbd3 RNA compared to uninjected embryos. RNA from TG and NTG GV stage oocytes was also used for quantitative RT-PCR. (C) A graphical representation of real-time quantitative RT-PCR experiments demonstrating reduced Mbd3 RNA levels in TG oocytes. TG and NTG bars represent the ratios of Mbd3 to H2afy2 levels (a H2 histone expressed in early embryos) derived from crossing points. The ratios are normalized to the levels in NTG embryos (shown as 1), and are the averages of three experiments. TG oocytes have only 15% of the Mbd3 RNA compared to NTG oocytes. (D) RT-PCR analysis for Mbd2 demonstrates targeting specificity for Mbd3 because no reduction in Mbd2 mRNA was seen. (E) Embryos were subjected to immunocytochemistry with the anti-MBD3 antibody as described in Figure 1B. MBD3 protein levels are greatly reduced in both dsMbd3 injected embryos (iv) and TG embryos (viii) when compared to dsGfp injected (ii) and NTG (vi) controls. Embryos were also treated with SytoX (i, iii) or DAPI (v, vii) to show the nuclei of the embryos. DsGfp injected embryos are shown in i, ii; dsMbd3 injected embryos are shown in iii, iv; with NTG (v, vi) and TG blastocysts (vii, viii) shown in the bottom panels.

To determine if MBD3 was required for imprinted gene expression, we employed two RNAi approaches (injection and TG RNAi) that reduce maternal stores of RNA, but could also persist to reduce newly transcribed zygotic Mbd3 messages (Figure 2A). In the first method (injection RNAi), in vitro transcribed dsRNA (dsMbd3 or control dsGfp) was injected into one-cell embryos. Embryos were then cultured to the blastocyst stage and collected for analysis. An additional group of uninjected embryos was also cultured to control for culture conditions. The second RNAi approach (TG RNAi) utilized a transgene to reduce Mbd3 RNA levels. The transgene employs the zona pellucida 3 *(Zp3)* promoter, which drives expression of linked sequences in growing oocytes [21]. The promoter is upstream of an *Mbd3* inverted repeat that generates an approximately 510-bp dsRNA that is identical to that used for the injection RNAi experiments.

Both RNAi strategies resulted in reduced Mbd3 RNA and protein levels. The RNA levels were examined by semi-quantitative reverse transcriptase PCR (RT-PCR) and real-time RT-PCR using *Mbd3*-specific primers and *Gapdh* primers for normalization (Figure 2B and 2C). Injection RNAi embryos were assessed at the blastocyst stage for RNA and protein. On average, blastocysts used for these experiments had only 35% of Mbd3 RNA compared to control embryos (Figure 2B). RNAi-treated embryos were assayed for Mbd2 RNA levels (Figure 2D) as *Mbd2* encodes the family member that is most closely related to *Mbd3* [22]. Mbd2 levels were unchanged in the dsMbd3-injected embryos, demonstrating that dsMbd3 is both effective and specific. RNAi-treated blastocysts were also assayed by immunocytochemistry with an anti-MBD3 antibody to determine protein levels at the blastocyst stage. dsMbd3-injected embryos had very little MBD3 protein compared to control embryos at the blastocyst stage, demonstrating that the MBD3 protein is labile enough to respond to dsRNA injection during this preimplantation period (Figure 2E).

**Figure 3:**
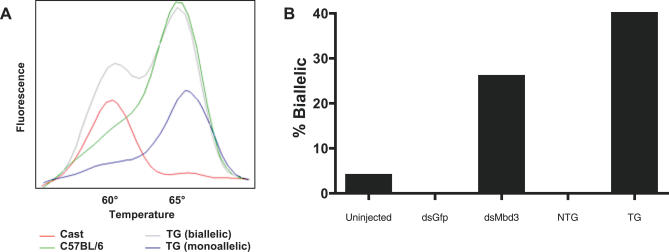
*H19* Is Derepressed at the Paternal Allele after Mbd3 RNAi One-cell C57BL/6J X B6(CAST7) embryos were injected with dsRNA, cultured to the blastocyst stage, and collected singly for RNA extraction. Similarly, TG and NTG embryos were either cultured as above or collected from mothers at 3.5 dpc. Real-time RT-PCR with allele-specific hybridization probes was used to determine *H19* expression from each allele, as shown in (A). Shown are the melting curves of the control samples along with a biallelic TG and monoallelic TG sample (the melting curves for the ten other samples processed at the same time are omitted for clarity). The red curve is the melting curve of a Cast control (probe melts off at 60 °C) and the green represents a C57BL/6J control (probe melts off at 65 °C). The grey curve is one of the biallelic TG blastocysts, with two peaks corresponding to the paternal Cast allele and the maternal C57BL/6J allele. The blue curve is a TG sample that is monoallelic for *H19* (maternal C57BL/6J allele only). (B) *H19* is biallelically expressed in 26% (9/35) of dsMbd3 injected embryos. This is significantly different from controls (*p* < 0.01 compared to uninjected [2/46] and *p* < 0.01 compared to dsGfp injected [0/28]). Most control embryos (uninjected and dsGfp) express only the maternal *H19* allele. In the *Zp3-dsMbd3* TG embryos, 40% (15/38) of TG embryos show biallelic expression of *H19*. None (0/5) of the NTG controls showed biallelic expression.

For the TG RNAi experiments, 11 TG lines were generated. Each line was assessed for levels of Mbd3 RNA at the GV stage of oogenesis, a time following the initiation of transgene expression. The lines showed a range of RNAi efficacy from 14% of control levels in the best line, line 37, (normalized to Gapd and compared to nontransgenic (NTG) controls as described above) to 100% of Mbd3 RNA in poor lines. The experiments described here were conducted using line 37 (Figure 3C), the best line in terms of RNA and protein depletion. Similar to the injection RNAi embryos, TG RNAi embryos showed a severe reduction in MBD3 protein at the blastocyst stage (Figure 2E). Together, these experiments revealed that the RNAi that targets Mbd3 is effective in reducing MBD3 protein levels when administered in the growing oocyte or at the one-cell stage.

### Loss of *H19* Imprinted Gene Expression in RNAi-Treated Embryos

With RNAi effectively and specifically reducing Mbd3 RNA and protein during the preimplantation period, these embryos could be used to determine if Mbd3 is required for proper imprinted gene expression during this time. Only a small group of imprinted genes, including the maternally expressed *H19* gene and the paternally expressed *Snrpn* gene, are first expressed and imprinted during the preimplantation period. As *H19* transcription commences at the late blastocyst stage, blastocysts from both injection RNAi and TG RNAi were examined for changes in expression from the normally silent paternal allele. An allele-specific real-time RT-PCR assay enables simultaneous examination of both parental alleles, thereby allowing for an assessment of paternal allele activation in single blastocysts [23]. In blastocysts derived from injection of one-cell embryos with dsRNA, a quarter of the embryos showed biallelic *H19* expression whereas only 4% of uninjected blastocysts and no dsGFP-injected blastocysts exhibited biallelic *H19* expression (Figure 3). Blastocysts from line 37 showed a similar but more robust effect; 40% of blastocysts derived from TG mothers exhibited biallelic *H19* expression whereas blastocysts from NTG mothers expressed only the maternal *H19* allele (Figure 3). In both sets of RNAi experiments, the ratio of paternal expression to total *H19* expression varied from 15%–55%. It should be noted that although the imprinting of *H19* is closely coordinated with that of the linked and oppositely imprinted *Igf2* gene, expression of *Igf2* is not evident until after implantation. Thus, the role of MBD3 in the regulation of *Igf2* could not be assessed using this RNAi system. Nevertheless, these experiments demonstrate that Mbd3 is required for proper imprinted expression of *H19* at the blastocyst stage.

RNAi-treated embryos were also assayed for the few other imprinted genes expressed at this stage. All embryos were assayed for *Snrpn* gene expression*,* but no change in *Snrpn* imprinting was observed, consistent with the absence of MBD3 binding in the *Snrpn* imprinting control region as determined by ChIP (unpublished data). TG RNAi animals were also assayed for *Peg3*, *Gtl2,* and *Atp7a*. *Peg3* is an imprinted gene on Chromosome 7 and is paternally expressed at the blastocyst stage (M. Mann and MSB, unpublished data). *Gtl2* is an imprinted gene on Chromosome 12 and is maternally expressed at the blastocyst stage [23]. *Atp7a* is located on the X chromosome and is silenced on the paternal allele due to imprinted X-inactivation in the preimplantation embryo [24]. No changes in expression of *Peg3* and *Gtl2* (in all embryos) and *Atp7a* (in female embryos) were observed in TG embryos (unpublished data). These results suggest that MBD3 protein is not required for all imprinted gene expression at the blastocyst stage, and may also indicate that the silent alleles at these loci employ other MBD family members to confer silencing of the inactive allele.

### Loss of NuRD Repressive Complex with Reduction of MBD3

**Figure 4:**
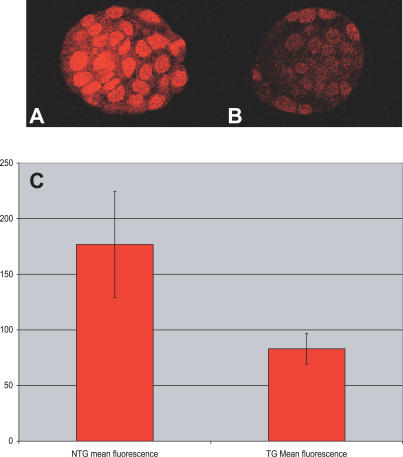
The Mi-2/NuRD Complex Protein MTA2 Is Reduced in Mbd3 RNAi Blastocysts (A, B) NTG (*n* = 4) (A) and Mbd3 RNAi TG (*n* = 16) (B) embryos were subjected to immunocytochemistry with the anti-MTA2 antibody. When Mbd3 is reduced in TG embryos, MTA2 protein is significantly reduced (compare [A] to [B]). (C) Measurement of mean nuclear fluorescence using ImageJ software of confocal images shows that the mean fluorescence of TG embryos have on average 47% of the nuclear fluorescent MTA2 signal compared to NTG samples.

Biochemical experiments indicate that MBD3 is an integral member of the chromatin-remodeling complex Mi-2/NuRD, and recent data suggest that in the absence of MBD3, this complex is unstable [25]. To determine whether this complex is intact in Mbd3 RNAi embryos, blastocysts from NTG (Figure 4A) and TG mothers (Figure 4B) were stained with an MTA-2-specific antibody. Consistent with the result seen with Western blotting of MTA2 in *Mbd3* null ES cells [25], Mbd3 RNAi TG blastocysts showed more than a 50% reduction in average MTA-2 fluorescence levels in blastocyst nuclei (Figure 4C), a statistically significant difference (*p* < 0.001). The requirement for MBD3 for *H19* imprinted expression and the accompanying reduction in MTA-2 levels strongly implicates a role for the Mi-2/NuRD complex in repression at this imprinted locus.

### Loss of *H19* Methylation in Mbd3–Depleted Embryos

**Figure 5:**
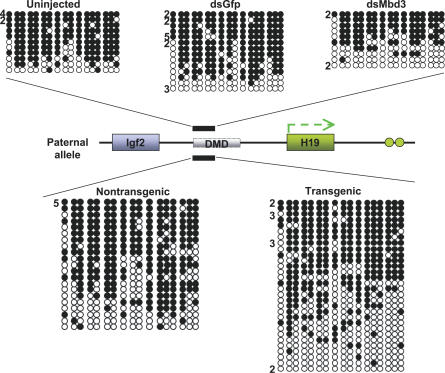
Reduction of Mbd3 in Embryos Results in a Loss of Methylation at the *H19* DMD One-cell C57BL/6J X B6(CAST7) embryos were injected with dsRNA, cultured to the blastocyst stage, and collected in pools of five blastocysts (top). Similarly, TG and NTG embryos were isolated at the one-cell stage, cultured to the blastocyst stage, and collected in pools of five (bottom). Bisulfite mutagenesis was performed on agarose-embedded pools of five blastocysts. CpGs (17) were assayed in the 5′ half of the DMD as indicated by the black line in the diagram in the center. Each line of circles represents a single DNA strand with the number to the left of the line corresponding to the number of times this pattern was seen. Each circle represents a single CpG. If the CpG was methylated, the circle is filled. Those strands with less than half of the CpGs methylated are considered hypomethylated. In control embryos, only a few hypomethylated strands are recovered (13% and 17% for uninjected and dsGFP-injected blastocysts, respectively), but in dsMbd3-injected embryos, 42% of the paternal strands are hypomethylated. TG RNAi blastocysts showed similar results with 42% of the paternal strands hypomethylated compared to 16% of the strands in NTG embryos. Only paternal strands are shown, as all sequenced maternal strands were unmethylated as expected. Although its expression also depends on the DMD, *Igf2* is not expressed at this stage.

The relaxation of paternal *H19* expression in Mbd3 RNAi blastocysts suggested that there might be changes in DNA methylation at the paternal DMD. To address this question, several pools of five blastocysts from RNAi-treated animals and controls were collected and DNA methylation changes in the *H19* DMD examined using bisulfite methylation assay. After the pools were subjected to bisulfite mutagenesis, PCR products were obtained from the 5′ portion of the DMD, cloned, and sequenced to determine the DNA methylation status of individual DNA strands (Figure 5). C57BL/6 (maternal) and *Mus musculus castaneus* (paternal) DNA polymorphisms were used to distinguish the two parental alleles. The 5′ DMD contains 17 CpGs over 426 bp, and strands that lacked nine or more methylated CpGs were considered hypomethylated.

A reduced number of DNA methylated strands was seen in at the *H19* DMD in RNAi-treated embryos when compared to controls. DsMbd3-injected embryos had 42% hypomethylated paternal strands compared to 13% and 17% for uninjected and dsGfp-injected controls, respectively (Figure 5). Similarly, pools from TG RNAi embryos had 42% hypomethylated paternal strands whereas NTG control pools contained only 16% hypomethylated paternal strands (Figure 5). A *Snrpn* PCR product was also obtained from these embryos and sequenced, but the normally methylated maternal alleles from dsMbd3-treated embryos did not differ from controls (unpublished data). These results suggest that MBD3 is required to maintain the paternal methylation mark during the preimplantation period at the *H19* locus.

The absence of Mbd3 in preimplantation embryos leads to both biallelic expression of *H19* and loss of methylation at the normally methylated paternal allele at the blastocyst stage. Since the RNAi effect is transient, we analyzed later stage embryos (7.5 days post coitus [dpc]) to determine if *H19* expression and DNA methylation defects are maintained. Both TG (*n* = 7) and NTG (*n* = 9) embryos expressed *H19* exclusively from the maternal allele. Likewise, both TG and NTG 7.5-dpc embryos exhibited a hypermethylated DMD on the paternal allele. Presumably, Mbd3 RNA and protein levels rise soon after the blastocyst stage. These results with later embryos suggest that either reduction of Mbd3 mRNA and protein has only a transient effect that can be repaired or that embryos with serious imprinting problems fail to develop further.

### Mbd3 RNAi Blastocysts Have Fewer Cells Than Control Embryos

During the course of these experiments, we noted that some dsMbd3-injected blastocysts were smaller than both uninjected and dsGfp-injected control blastocysts. To investigate this observation further, control and experimental blastocysts were collected to determine if the Mbd3 RNAi embryos had fewer cells. These experiments revealed that RNAi embryos have significantly fewer cells than control embryos (Table 1). For injection RNAi, uninjected blastocysts have an average of 97 cells, dsGFP injected embryos have an average of 87 cells, and dsMbd3 RNAi blastocysts have an average of 74 cells per embryo (*p* < 0.01). TG RNAi blastocysts (77.5 cells) also have fewer cells than NTG control blastocysts (93.8 cells; *p* < 0.05).

**Table 1:**
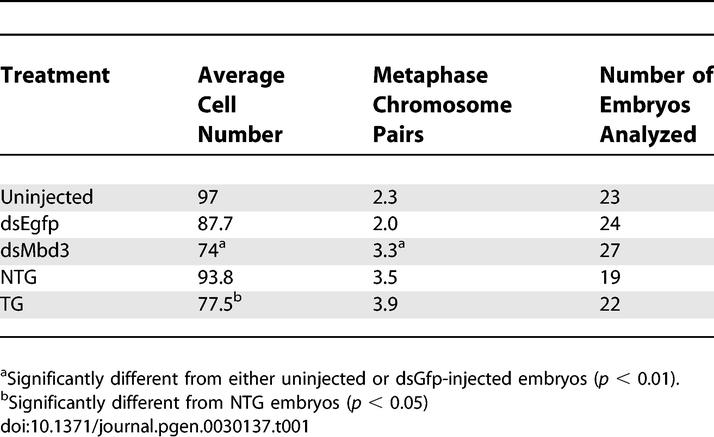
Cell Numbers of Blastocysts Following RNAi

The dsMbd3 embryos did not appear grossly different from control embryos other than the noticeable difference in size, although the incidence of hatching of dsMbd3-treated blastocysts was reduced (unpublished data). DAPI or propidium iodide staining revealed the nuclei to be normal in size and morphology and the blastocysts did not show any evidence of increased apoptosis as determined by TUNEL assay (unpublished data). The DAPI staining revealed that Mbd3-injected RNAi embryos but not TG RNAi embryos have a significantly increased number of metaphase chromosome pairs (Table 1). RNAi-treated embryos were also assayed by immunocytochemistry for expression of markers of the inner cell mass (OCT4) and trophoblasts (TROMA-1). DsMbd3-treated embryos showed expression of both of these markers at the blastocyst stage, suggesting that RNAi blastocysts form both of these tissue types at an appropriate time. Interestingly, Kaji et al. saw analogous results with smaller 5.5-dpc *Mbd3* −/− null embryos even though blastocysts were normal size [26]. Given that the *Mbd3* −/− embryos were derived from *Mbd3* +/− parents, the null embryos had maternal stores of MBD3 protein and RNA, which is lacking in our RNAi embryos and which would support normal cell division longer than RNAi-treated embryos. Together, these data indicate that MBD3 is required for proper cell division timing during development, but that eventually these embryos become blastocysts with both inner cell mass and trophoblast cells.

## Discussion

There are two times in development when the mammalian genome undergoes demethylation [27,28]. The first is during primordial germ cell migration and colonization of the genital ridge, when the entire genome, including imprinted genes, is demethylated. The second time is during preimplantation development, when the majority of the genome undergoes demethylation. Whereas the paternal genome undergoes almost immediate, presumably active, demethylation after fertilization [29–32], the maternal genome is demethylated gradually, due to a loss of maintenance DNA methylation, during cleavage divisions [33]. Most methylated sequences are demethylated during these two events, but imprinting control regions escape this demethylation. Specifically, for the paternally methylated *H19* DMD, this region must survive the active demethylation and then stay methylated in subsequent stages during passive demethylation [34,35]. How these processes occur has remained completely unknown up to this point. We propose that MBD3 is intimately involved in these processes at the *H19* locus during preimplantation development.

We initiated our studies with MBD3 for several reasons: it is expressed during this critical period of preimplantation development, *Mdb3* knockouts display an early embryonic lethal phenotype [17], and MDB3 is associated with chromatin at the *H19* DMD (Figure 1C). Two RNAi approaches allowed us to reduce Mbd3 RNA and protein from oocytes and preimplantation mouse embryos. The RNAi strategy for depleting target RNA has several advantages over traditional gene targeting experiments: RNAi allows for (1) reduction in maternal RNA and protein that persists in traditional knockouts, (2) production of a hypomorphic allelic series, and (3) easy use of hybrid strains for allelic gene expression analysis.

Using two types of RNAi approaches—injection of dsRNA and TG RNAi—we observe the same effects on the imprinted expression and DNA methylation of *H19*. Both methods proved effective at reducing Mbd3 RNA and protein. In RNAi embryos, but not control embryos, *H19* is biallelically expressed in a significant fraction of blastocysts examined (40% of TG RNAi and 26% of injection RNAi blastocysts). These results demonstrate that MBD3 is required for proper imprinted expression of *H19*.

Our initial hypothesis was that an MBD would bind to the methylated DMD and recruit repressive complexes to silence the paternal allele, but it is important to note that MBD3 fails to bind methylated substrates as assayed by in vitro DNA binding assays, likely due to a phenylalanine instead of a critical tyrosine in its MBD motif [22,36]. Whereas in vitro experiments suggest that MBD3 does not bind directly to the methylated DMD, our ChIP results indicate that MBD3 is directly associated with chromatin at the *H19* DMD. Similarly, chromatin immunoprecipitation experiments demonstrate that MBD3 interacts with the methylated maternal allele of the imprinted gene *Zrsr1* in adult mouse livers [20]. Furthermore, MBD3 is an integral member of the chromatin-remodeling enzyme Mi-2/NuRD, and recent data suggest that in the absence of MBD3, this complex is unstable [25]. The reduction of MTA-2 levels in Mbd3 RNAi embryos suggests that the amount of repressive NuRD complexes is reduced in RNAi embryos, and that in the absence of these complexes, *H19* can be expressed from the paternal allele. These data suggest an important role for the NuRD complex in imprinted gene expression. Given the previously described genetic and biochemical interactions between MBD2 and MBD3 [17,37], MBD2 may also have a role in imprinted gene repression. However, *Mbd2*−/− embryos have been examined and shown to have proper expression of several imprinted genes including *H19* [17]. Experiments to reduce simultaneously levels of MBD3 and other MBDs should uncover any such role for MBD2 that may have been compensated for by the presence of MBD3.

Although *H19* is biallelically expressed in a significant number of Mbd3 RNAi blastocysts, not all blastocysts exhibit such a loss of imprinting. Likewise, the DMD is not completely demethylated on every strand, although there are strands that were completely demethylated (Figure 5). There are several possible explanations for this result. First, RNAi may not completely ablate MBD3 function. Quantification of RNA levels indicated a fairly robust reduction in Mbd3 RNA levels (Figure 2), and a nearly complete loss of protein, as determined by immunocytochemistry, but, with the technology currently available, it is difficult to determine the effective loss of MBD3 at the level of chromatin, where it presumably acts. Second, even if robust reduction of MBD3 is attained, MBD3 is one of several family members that are expressed in preimplantation embryos, and these other family members might provide compensatory activity in the absence of MBD3. Finally, the timescale of this experiment may not provide adequate time for MBD3 protein to turn over completely, and for the results of the RNA reduction by RNAi to be completely realized.

We examined expression of a handful of other imprinted genes in RNAi embryos, but none showed derepression of the silent allele, suggesting that MBD3 is not involved in their repression. In this case, other MBDs might be required for their imprinted expression. Alternatively, the presence of other MBDs might be sufficient to compensate for the reduction in MBD3 at these loci, but not at *H19*. By reducing several MBDs in concert by RNAi, such compensatory mechanisms might be uncovered. Also, *H19* may be especially sensitive to perturbation of imprinting cofactors and the paternal allele may be derepressed with the slightest insult to the repressive complex, whereas other imprinted genes may be able to make do with reduced MBD3 protein. Other types of experiments with longer-lived effects such as knockout alleles might be better suited to uncovering these effects as well as determining if MBD3 is required for the imprinting of genes such as *Igf2* that are expressed later in development*.* Finally, the continued discovery of new proteins with an affinity for methylated DNA might eventually uncover different mechanisms for the recognition and silencing of methylated sequences.

Our data suggest that MBD3 is required for maintenance of DNA methylation at the *H19* DMD during preimplantation development. Similar to the result in which a portion of embryos exhibit biallelic expression, a significant fraction of DNA strands are demethylated. For the reasons outlined above, the loss of MBD3 at this locus may not be complete or its effects may not be complete at the blastocyst stage. The partial loss of DNA methylation and accompanying loss of repression at *H19* suggests there may be a threshold for DNA methylation loss and concomitant paternal expression of *H19*. Indeed, the percentage of hypomethylated strands is similar to the number of embryos with biallelic expression of *H19*. Our data reporting loss of methylation at the *H19* DMD in the absence of MBD3 uncovers a new role for MBD proteins: protection of methylated regulatory sequences from DNA demethylation during preimplantation development. This role may not have been predicted by the MBD3 protein sequence, but it is clear that methylated imprinted regions must both be recognized and maintain their parental marks during this developmental time period. It is an interesting finding that a repressive complex that recognizes a differentially methylated region would also be responsible for maintaining that difference during development.

In conclusion, development of embryos that lack MBD3 is perturbed, whether MBD3 is removed transiently by RNAi (this study) or by a traditional deletion of the *Mbd3* locus [17]. In the RNAi experiments, imprinted *H19* expression is disturbed along with loss in DNA methylation on the paternal allele. In addition, the Mbd3 RNAi blastocysts are smaller and slower to hatch than controls, suggesting delays in development and perturbed cell cycles. *Mbd3* −/− animals die during the peri-implantation period, but the phenotype had not been extensively described until recently [17,26]. Kaji et al. (2006) reported that *Mbd3*−/− ES cells did not exhibit any cell cycle delay when the ES cells were examined by flow cytometry [25], but that 5.5-dpc embryos have reduced cell numbers [26]. These results suggest that the smaller Mbd3 RNAi embryos may be due to a developmental timing defect rather than a cell-cycle delay. TUNEL assays did not detect an increase in the number of cells undergoing apoptosis between RNAi embryos and controls, suggesting that RNAi embryos are not smaller due to increased cell death (unpublished data). Furthermore, both inner cell mass and trophoblast cells are present, suggesting no defects in the first preimplantation lineage decisions. Consistent with delays in cell division that we observe, cells deficient for *Hells,* a member of the SNF2 family of chromatin remodelers that is also a global regulator of DNA methylation, have defects in DNA methylation and are slow in completing mitosis [38]. Thus, the hypomethylation observed in Mbd3 RNAi embryos might similarly lead to problems in cell division in embryos that may not be observed in cultured ES cells. Finally, in both our study and in the studies of *Mbd3*−/− ES cells, loss of Mbd3 negatively affects the Mi-2/NuRD complex [25]. Consequently, even a transient MBD3 loss could lead to a global disruption of silencing carried out by this complex beyond the perturbation we report at the *H19* locus. A microarray analysis of global transcript profiles might uncover more genes regulated by this complex or genes specifically responsible for the other phenotypes we see beyond imprinting defects. Our results, thus, uncover an essential role for MBD3, and the Mi-2/NuRD complex, in regulating the imprinted *H19* locus, and provide tools to explore additional roles for MBD3 in genome-wide transcriptional regulation.

## Materials and Methods

### Mice.

For dsRNA injections, oocytes and embryos were obtained from either CF1 (Harlan, http://www.harlan.com/) females mated to CF1 males (for embryo assays) or from C57BL/6J (The Jackson Laboratory, http://www.jax.org/) females mated to B6(CAST7) males (for allelic assays) [23]. Embryos from TG animals were obtained by mating TG females to B6(CAST7) or to B6(CAST7P12X) males. This latter strain has *Mus musculus castaneus* Chromosomes 7, 12, and X in a C57BL/6 background (M. Mann, J. Mager, C. Krapp, and MSB, unpublished data). All experiments were conducted with the approval of the Institutional Animal Care and Use Committee at the University of Pennsylvania.

### Production of dsRNA.

Primers KR1 (5′-CTATGGAGCGGAAGAGGTGGGA-3′) and KR3 (5′-CAGGCCCACTCCCTGCAGGC-3′) were used to amplify a 510-bp fragment corresponding to bp 1 to 510 of *Mbd3* with Ready-to-Go PCR beads (Amersham http://www.amersham.com/) from ES cell cDNA. PCR was performed for one cycle of 94 °C for 2 min followed by 35 cycles of 15 s at 94 °C, 10 s at 55 °C, and 15 s at 72 °C, and one cycle of 72 °C for 15 min. The PCR product was cloned using the Gateway BP reaction (Invitrogen, http://www.invitrogen.com/) into the pDONR201 vector to give the pKR3.03 entry clone. This plasmid was combined in a Gateway LR reaction with the L4440 Gateway C plasmid that contains two T7 promoters flanking the recombination sites to give pKR3.04. For in vitro transcription, pKR3.04 was used as a template for PCR with primers L4440F (5′-AGCCGAACGACCGAGCGC-3′) and L4440R (5′-TGCAAGGCGATTAAGTTG-3′) that flank the T7 promoters. The PCR product was used as a template for a single T7 reaction that produces both sense and antisense RNA. For in vitro transcription of *Gfp*, a *Not*I -*Hind*III fragment from pEGFPN-2 (Clontech, http://www.clontech.com/) was also cloned into the L4440 Gateway C plasmid. Each 100-μl in vitro transcription reaction contained 5–10 μg of PCR template, 1× T7 transcription buffer, 30 mM rNTPs, and T7 enzyme mix (RiboMAX T7; Promega, http://www.promega.com/). Samples were incubated at 37 °C for 2 h, after which the template was digested with RQ1 RNase-free DNaseI (Promega) for 15 min at 37 °C. Enzymes were removed with two phenol:chloroform:isoamyl alcohol (25:24:1) and two chloroform:isoamyl alcohol (24:1) extractions and RNA was recovered from the aqueous phase by two ethanol precipitations with 1/10 volume of 10 M ammonium acetate. The dsRNA was resuspended in 100 μl of RNase-free H_2_O, and its concentration was estimated on a 1% agarose gel by comparing to a mass ladder standard (Invitrogen).

### dsRNA injections.

Oocytes and preimplantation embryos from CF1 or C57BL/6J mothers were microinjected with approximately 10 pl of 0.25 μg/μl dsRNA. Oocytes were cultured in CZB medium for 24 h at 37 °C in an atmosphere of 5% CO_2_. One-cell embryos were isolated at 0.5 dpc and cultured to the blastocyst stage (96 h) in KSOM with amino acids (KSOM + AA) at 37 °C in an atmosphere of 5% CO_2_, 5% O_2_, and 90% N_2_ [39]. DsGFP-injected and uninjected control embryos were cultured alongside dsMbd3-injected embryos to control for culture conditions.

### TG mice.

To generate the Zp3-dsMbd3 TG construct, an inverted repeat was cloned into pRNAi-Zp3–1 [40]. First, primers KR1 and KR3 were used to generate a 510-bp PCR fragment that was cloned into pCR2.1 using the TOPO TA kit (pKR3.18). The inverted repeat was generated by excising an XhoI–XbaI *Mbd3* fragment from pKR3.03, and ligating it into XhoI- and XbaI-digested pKR3.18, creating the inverted Mbd3 repeat plasmid pKR3.19. The inverted repeat was then excised with SpeI and ligated into pRNAi-Zp3–1 that had been digested with XbaI, to give pMoZp3-dsMbd3.

TG animals were generated as previously described [41]. Animals were genotyped by PCR assay for *Gfp* as previously described using DNA isolated from tail or ear clippings as previously described [21]. This was confirmed by Southern blot using a *Gfp* probe (unpublished data). TG lines were maintained on a C57BL/6J background. Line 37 Mbd3 RNAi TG females described in this study are fertile.

### Isolation of germinal vesicle stage oocytes and preimplantation embryos.

Germinal vesicle (GV) stage oocytes were isolated from females at least 6 wk of age, as previously described [21]. For later-stage embryos, females, at least 6 wk of age, were superovulated by injection of 7.5 IU of pregnant mare serum gonadotropin (Calbiochem, http://www.emdbiosciences.com/) and 44–48 h after injection, injected with 5.0 IU of human chorionic gonadotropin (hCG) and set up for matings with stud males; females were checked for copulatory plugs the following morning (0.5 dpc). Fertilized zygotes were collected at 0.5 dpc, two-cell embryos at 1.5 dpc, and blastocysts at 3.5 dpc. Embryos were isolated in phosphate-buffered saline containing 3 mg/ml polyvinylpyrrolidone (PBS/PVP, Calbiochem). For isolation of RNA, embryos were transferred to 100 μl of lysis buffer (Dynal, http://www.invitrogen.com/). Embryos used for bisulfite mutagenesis were transferred in several microliters of PBS/PVP to a 1.5-ml tube. One-cell embryos were cultured to the blastocyst stage in KSOM + AA as described above.

### RNA isolation, reverse transcription, and PCR.

Poly-A^+^ mRNA was isolated from oocytes and preimplantation embryos using the Dynabead RNA Isolation Kit (Dynal) according to manufacturer's instructions and reverse transcribed for 60 min at 42 °C followed by 10 min at 95 °C as described previously [23].

All PCRs were carried out using Ready-to-go-PCR beads in a final volume of 25 μl and included 0.1 μCi of [α-^32^P]-dCTP (New England Nuclear [NEN], http://las.perkinelmer.com/). The reaction conditions were such that the amount of product was in the linear region of semi-log plots of the amount of product versus cycle number [42]. Mbd3 was amplified from 1.5 oocyte (or blastocyst) equivalents using a final concentration of 0.6 μM of each primer, KR15 (5′-TGTCAGCCATTGCGAGTGCTC-3′) and KR18 (5′-CTACACTCGCTCTGGCTCCGG3–3′). These primers span an intron. Mbd3 PCR was carried out for one cycle of 94 °C for 2 min followed by 27 cycles for oocytes (25 cycles for blastocysts) of 15 s at 94 °C, 10 s at 63 °C, and 20 s at 72 °C. Gapd was amplified from 0.5 oocyte (or blastocyst) equivalents as described previously [21]. Samples for developmental profiles were spiked with 0.125 pg/embryo equivalent of rabbit α-globin (Invitrogen). Rabbit α-globin was amplified from 0.5 oocyte equivalents as described previously [43]. Mbd3 and Gapd were amplified from the same samples to determine efficacy of RNAi treatments using cycle numbers in the linear range for each as described above. The Mbd3 levels were then normalized to Gapd levels from the same samples. Quantification was performed using the ImageQuant 5.2 program (Molecular Dynamics, http://www6.gelifesciences.com/). Results with TG RNAi samples were confirmed by real-time RT-PCR using the *Mbd3* and *H2afy3* light cycler primers (Table S1) using sequence-specific detection probes (Universal Probe Library; Roche, http://www.roche.com/) as described by the manufacturer on a LightCycler Instrument (Roche).

### Allele-specific expression analysis of blastocyst stage embryos.

*H19* and *Snrpn* expression assays were conducted on cDNA using the LightCycler Real Time PCR System (Roche Molecular Biochemicals) as described [23,44]. *Gtl2* and *Peg3* RT-PCR expression assays were conducted on cDNA from single blastocysts and included 0.1 μCi of [α-^32^P]-dCTP (NEN). The data were quantified by phosphorimager analysis following allelic restriction digests [44].

### Immunofluorescence.

Oocytes or preimplantation embryos were fixed in 3.7% paraformaldehyde, pH 7.5, for 60 min at room temperature or overnight at 4 °C, washed two to three times in PBS/PVP, and stored at 4 °C in PBS/PVP. For staining, samples were permeabilized for 15 min in freshly prepared PBS containing 0.25% Triton-X100, washed three times in PBS/PVP, then blocked for 1 h in 5% donkey serum/0.1% fish gelatin/0.2% Tween-20/PBS. Samples were then incubated overnight with polyclonal goat antibody against an MBD3 peptide found in both mouse and human MBD3 (Santa Cruz Biotechnology, http://www.scbt.com/) diluted 1:50 in blocking solution. The specificity of the antibody was confirmed by loss of nuclear signal after blocking with the peptide the antibody was raised against (Santa Cruz Biotechnology) and further validated by the presence of a 34-kDa band on a Western blot of HeLa cell extracts. Blastocysts contain too little protein for visualization of MBD3 or MTA-2 by Western blotting. After three 15 min washes in 0.2% Tween-20/PBS, samples were transferred to a 1:500 dilution of donkey Cy3-conjugated anti-goat IgG (Jackson ImmunoResearch Laboratories, http://www.jacksonimmuno.com/) and 2 μg/ml DAPI (Sigma, http://www.sigmaaldrich.com/) in blocking solution for 60 min. Samples were washed three times for 15 min. Blastocysts were equilibrated in increasing amounts of Vectashield (Vector, http://www.vectorlabs.com/) to maintain shape and maximal fluorescence. Finally, oocytes or embryos were mounted on a glass slide and visualized with a confocal microscope (Leica, http://www.leica.com/). All incubations were at room temperature unless otherwise noted. In order to count nuclei, *z*-stacks of the confocal images through each blastocyst were collected, with 2.5 μm between each *z-*plane. Stacks were assembled into movies in NIHImage (http://rsb.info.nih.gov/nih-image/), and nuclei were counted manually in each plane, taking care to note which nuclei had been counted in the previous plane. To determine mean fluorescence, ImageJ (http://rsb.info.nih.gov/ij/) was used to draw a region of interest inside each of five nuclei per embryo in the middle slice of confocal stacks and to measure the mean pixel intensity in the region. The values for TG and NTG embryos were averaged and graphed in Excel (http://office.microsoft.com/) to obtain the data in Figure 4.

### Southern analysis.

Ten micrograms of isolated DNA were digested with StuI, separated on a 0.8% agarose gel, transferred to a nylon membrane, and probed as previously described [41]. The *Gfp* probe fragment was isolated from pEGFP-N1 (Clontech) and labeled with [α-^32^P]-dCTP (NEN). Blots were analyzed using a Phosphorimager (Molecular Dynamics).

### Bisulfite mutagenesis.

Pools of five blastocysts were embedded in approximately 10 μl of molten 2% low-melting point SeaPlaque agarose (BioWhittaker Molecular Applications, http://www.lonzabioscience.com/), and treated as previously described [21]. To prepare DNA for PCR, pellets were resuspended in an appropriate amount of water, and melted 5 min first at 65 °C and then at 80 °C. Blastocyst equivalents (2.5) of the mutagenized DNA were used for each PCR and products were cloned and sequenced as described previously [21]. Six or more clones were sequenced for each PCR. Strands from a PCR that contained an identical pattern of methylated CpGs and that could not be distinguished from other strands by polymorphisms were only counted once.

### Chromatin immunoprecipitation assays.

ChIP assays were carried out using the Chromatin Immunoprecipitation Assay Kit (Upstate, http://www.upstate.com/) according to the manufacturer's instructions, with a few modifications. The ES cells used in these experiments were derived from blastocysts generated from crosses between C57BL/6 (B6) female mice and B6(CAST7P12X) male mice. The ES cells were grown on MEF feeder layers in 60-mm dishes, trypsinized, and replated on gelatinized plates to allow MEFs to attach for 1 h. The supernatants containing the ES cells were then collected in 50-ml conical tubes, crosslinked with 1% formaldehyde (Sigma) for 15 min at room temperature, and then the reaction was quenched with 0.125 M glycine for 5 min at room temperature. Following lysis, sonication was carried out using a Branson Sonifier 250 (http://www.sonifier.com/) at 30% output for four pulses of 10 s each. The sonicated cell supernatant was diluted 10-fold in ChIP dilution buffer (Upstate) and 1% of material was removed prior to the addition of antibodies (input). 80–100 μg of chromatin was used for each immunoprecipitation reaction with the following antibodies: anti-acetyl-Histone H3 (06–599; Upstate) and anti-MBD3 (ab3755; abcam, http://www.abcam.com/) [18,20]. For the final step, samples were resuspended in 30 μl of TE and 1 μl was used for each PCR. Input DNAs were diluted 10-fold. Three separate ChIP experiments were performed with the antibodies described.

### Allele-specific ChIP PCRs.

For allele-specific analysis, input and immunoprecipitation DNAs were amplified with primers specific to different regions at *H19, Snrpn,* and *Oct4* (see Table S1). Briefly, all PCRs were carried out with Ready-To-Go PCR beads (Amersham) using 0.3 μM of each primer and 0.1 μCi of [^32^P]-dCTP. A fraction of the PCR was used for each digest (see Table S1). Products were resolved on 7% polyacrylamide gels and the relative band intensities were quantified using ImageQuant (Molecular Dynamics). Real time PCR analysis was carried out on the ChIP samples using the LightCycler Real Time PCR System (Roche) to quantitatively assess the amount of chromatin bound in each ChIP experiment, and to determine the linear range of each ChIP PCR (unpublished data). Briefly, reactions were set up in triplicate using the Ready-To-Go PCR beads (Amersham), with a 5-min initial incubation with the TaqStart antibody (Clontech), followed by addition of 0.3 μM primers (see Table S1), 1× SYBR Green (Roche), and 1.5–3.0 mM MgCl_2_. Data analysis was performed using the Light Cycler version 4.0 software (Roche).

### Statistical analysis.

Statistical values for each dataset, with the exception of the ChIP data, were determined by the Fisher exact test using the GraphPad Prism statistics software suite (http://www.graphpad.com/prism/). For the ChIP data, average percentages (and standard deviations) of bound material for the ChIP experiments were calculated, and these means were statistically compared using a one-tailed *t*-test.

## Supporting Information

Table S1 Primers and Conditions for PCR Analysis (37 KB DOC)

### Accession Number

The National Center for Biotechnology Information (NCBI) GenBank (http://www.ncbi.nlm.nih.gov/sites/entrez?db=Nucleotide) accession number for *Mbd3* is AF120995.

## Abbreviations

ChIP: chromatin immunoprecipitation
DMD: differentially methylated domain
dpc: days post coitus
DsRNA: Double-stranded RNA
ES: embryonic stem
MBD: Methyl-CpG-binding protein
Mi-2/NuRD: nucleosome remodelling and histone deacetylase complex
NTG: nontransgenic
RNAi: RNA-mediated interference
RT-PCR: reverse transcriptase PCR
TG: transgenic
Zp3: zona pellucida 3
