# Supplementary material for: Maintenance of Paternal Methylation and Repression of the Imprinted H19 Gene Requires MBD3
Source: PLoS Genet. 2007 Aug 17;3(8):e137. doi: 10.1371/journal.pgen.0030137 (PMC1950162; doi:10.1371/journal.pgen.0030137)
Supplement: Supplementary file 1 [file pgen.0030137.st001.doc]

**Supplemental Table 1: Primers and conditions for PCR analysis**

| Region | Primers | Restriction Enzyme | Products |
| --- | --- | --- | --- |
| *H19* DMD  (allelic ChIP) | Rep3F2: CAGTTGTGTTTCTGGAGGG  Rep3R2: TAGGAGTATGCTGCCACC | Tsp45I | B6 110, 28  Cast uncut 137 |
| *H19*  (ChIP qPCR) | 4017F: CAGGACTCAAAGGAACATGCTAC  3594R: GCAATCCGTTTTAGGACTGCG | none | 398 bp |
| *Snrpn* UP  (ChIP) | SnUPCHIPs AATCTGTGTGATGCTTGCAATCACTTGG  SnUPCHIPs ATAGGATGCACTTTCACTACTAGAATCC | SmlI | B6 278, 142  Cast uncut |
| *Oct4*  (ChIP) | Oct4 F CCTCCGTCTGGAAGACACAGGCAGATAGCG  Oct4 R CGAAGTCTGAAGCCAGGTGTCCAGCCATGG | None, not allelic | 220 bp |
| *Mbd3* (Real time PCR) | Mbd3#69L AGGCACCACTGGACAAGG  Mbd3#69R CCTGTGCTACACTCGCTCTG | None  Probe #69 | 89 bp |
| *H2afy2* (Real time PCR) | H2afy2#72L GCGGTCACTGAGTACCTAGCA  H2afy2#18R GTTGAGTTCCTGGTCGTTGG | None  Probe #18 | 93 bp |
